# Variations in Velocity and Sensitivity of Electromagnetic Waves in Transmission Lines Configured in Model Piles with Necking Defects Containing Soils

**DOI:** 10.3390/s20226541

**Published:** 2020-11-16

**Authors:** Jung-Doung Yu, Sang Yeob Kim, Jong-Sub Lee

**Affiliations:** School of Civil, Environmental, and Architectural Engineering, Korea University, Seoul 136-701, Korea; jungdoung@gmail.com (J.-D.Y.); s3778@korea.ac.kr (S.Y.K.)

**Keywords:** bored piles, defect detection, electromagnetic wave, nondestructive evaluation, time domain reflectometer (TDR)

## Abstract

This study investigates variations in the velocity and sensitivity of electromagnetic waves in transmission lines configured in defective model piles for the detection of necking defects containing soil. Experiments are performed with model piles containing defects filled with different materials, such as air, sands, and clay. Five different types of transmission lines are configured in model piles. The electromagnetic waves are generated and detected using a time domain reflectometer. The velocity of electromagnetic waves is highest when the defect is filled with air, and it decreases with an increase in the water content. The velocity is lowest when the defect is filled with clay. The sensitivity of transmission lines for detecting defects decreases with an increase in soil water contents. The transmission line with a single electrical wire and epoxy-coated rebar exhibits the highest sensitivity, followed by that with three and two parallel electrical wires. Transmission lines with a single electrical wire and uncoated rebar and those with two parallel electrical wires wrapped with a sheath exhibit poor sensitivity when the defect is filled with clay. This study demonstrates that electromagnetic waves can be effective tools for detecting necking defects with wet and conductive soils in bored piles.

## 1. Introduction

A bored pile is a reinforced-concrete foundation that supports a superstructure by transferring vertical loads into the ground [1]. Bored piles are subjected to lateral loads by soil pressure, in addition to vertical loads [2]. Thus, the presence of a necking defect, which is a decrease in the cross-sectional area of the bored pile, can lead to unsatisfactory performance of the bored pile. The bored pile is typically constructed as follows: the ground is drilled to make a hole, a steel reinforcement cage is inserted into the hole, and concrete is poured into the hole. However, necking defects are occasionally introduced in the bored pile during or after the construction due to soil inclusion, poor concrete stiffness, intrusion of ground water, and slurry, among others [3]. It has been reported that 20.1% of bored piles are defective [4]. Thus, pile integrity tests for the detection of necking defects are necessary to ensure the safety of bored piles.

The coring examination is a conventional method employed to inspect necking defects in bored piles. Therein, a hole is drilled through the entire length of the bored pile. However, the coring method is time-consuming and expensive [5]. Furthermore, it is a destructive method that reduces the cross-sectional area of the bored pile. To overcome the limitations associated with the conventional destructive testing method, nondestructive testing (NDT) methods have been employed for detecting defects in bored piles. The sonic echo (SE) method is one of the widely used NDT methods for detecting necking defects in bored piles. In this method, stress waves are generated by impacting the head of the bored pile, and stress waves reflected from necking defects and/or the toe of the bored pile are then received by an accelerometer installed at the head of the bored pile. The defect locations are estimated using the velocity of stress waves reflected from necking defects. The SE method is quick, portable, and cost-effective [6,7]. However, the test effectiveness of the SE method is limited to bored piles with lengths of less than 30 times their diameter, because the propagation energy of stress waves leaks into the ground. The propagation energy of stress waves is significantly attenuated due to the impedance mismatch between the pile cap and bored pile. It is difficult to identify the defect locations due to multiple reflections in the case where two or more defects are introduced in the bored pile. Furthermore, horizontal locations of defects cannot be evaluated with the SE method [6,8,9,10,11,12]. Cross-hole sonic logging (CSL) is one of the most common NDT methods for detection of defects in bored piles. The CSL method requires two or more steel or polyvinyl chloride (PVC) access tubes, which are preinstalled alongside the reinforcement cage before casting the concrete. A source and receiver of the ultrasonic transducer are lowered inside separate access tubes and then simultaneously lifted while measuring ultrasonic waves. The defects are evaluated from the viewpoint of the attenuation and velocity of ultrasonic waves. The CSL method can be employed to detect necking defects in bored piles regardless of their length or diameter. In addition, the CSL method provides information on horizontal locations of defects and can simultaneously detect the locations of multiple defects. However, only defects that exist between access tubes are detectable with the CSL method. Moreover, defects are not detected if defect sizes are smaller than one-third of the interval between access tubes or one-tenth of a pile diameter. Access tubes reduce the cross-sectional area of the bored pile, which affects its bearing capacity [8,10,12,13]. Thus, a new NDT method for detecting necking defects in bored piles regardless of a pile length and diameter, reduction of cross-sectional area of the pile, and number, as well as direction of the defects, is required.

Recently, Lee et al. [14] suggested a novel and improved NDT method for inspecting necking defects in bored piles. In their study, small-scale laboratory experiments were conducted to demonstrate the suitability of electromagnetic waves for detecting necking defects in bored piles. The two-conductor transmission lines were configured with a reinforcement cage and electrical wires to guide the propagation of electromagnetic waves. Electrical wires with diameters of 2.9 mm were selected to avoid affecting the cross-sectional area of the bored pile. Consequently, both vertical and horizontal locations of necking defects were accurately estimated, despite multiple defects being introduced in the bored pile. The cross-sectional radii of the electromagnetic fields generated around transmission lines were below 10 cm. However, bored piles in the field are embedded in various types of soils, and necking defects can be filled with these soils. As the propagation of electromagnetic waves is affected by dielectric constants of surrounding materials, it is necessary to investigate the effects of soils on the electromagnetic wave propagation in bored piles. Lee et al. [15] pointed out that the influence of the dielectric constant on the propagation of electromagnetic waves in a rebar must be considered for the accuracy of the measurement. The propagation of electromagnetic waves is also affected by the electrical conductivities of surrounding materials. Since the electrical conductivity is related to electrical loss, conductive soils significantly attenuate electromagnetic waves. The reflection of electromagnetic waves in highly conductive soils is not observed due to leakage currents into soils [16,17,18,19]. Moreover, the spatial sensitivity of electromagnetic waves in soils is affected by the probe spacing, diameter, and coating thickness [20]. Thus, further studies are required to investigate the effects of soil water contents on the propagation of electromagnetic waves in bored piles.

The velocity and sensitivity of electromagnetic waves in transmission lines configured in bored piles are important factors for detecting necking defects. However, the velocity and sensitivity of electromagnetic waves in soil depend on the electrical properties of the soil. Thus, the type of soil filling the necking defects can have adverse effects on defect detectability. The proposed study aims to investigate variations in the velocity and sensitivity of electromagnetic waves in different types of transmission lines configured to detect defects in model piles with necking defects filled with soil. Experiments were performed with small-scale model piles; thus, the characteristics of electromagnetic waves propagating along long lengths were not investigated. Herein, small-scale model piles were constructed with defects filled with different materials, such as air, dry and wet sands, and clay. Five different types of transmission lines were configured in model piles to investigate transmission lines that are sensitive to necking defect detection. Necking defects were introduced around the main rebars of reinforcement cages. The velocity and sensitivity of electromagnetic waves in the five transmission lines configured in model piles were compared with respect to different soil types. The remainder of this paper presents the following sections: theoretical background, experiments, analysis and discussion, and summary and conclusions.

## 2. Theoretical Background

### 2.1. Transmission Line

**Figure 1:**
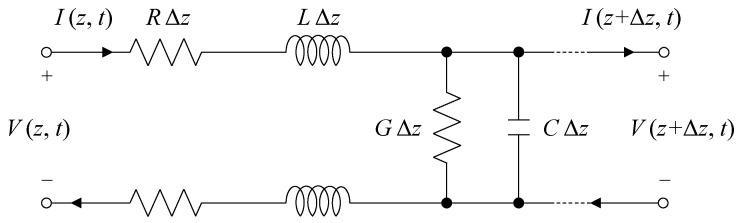
Equivalent circuit model of the differential length Δ*z* of a transmission line. *R*, *L*, *G*, and *C* denote resistance (Ω/m), inductance (H/m), capacitance (F/m), and conductance (S/m), respectively. *V* (*z*, *t*), *V* (*z* + d*z*, *t*), *I* (*z*, *t*), and *I* (*z* + d*z*, *t*) denote the instantaneous voltages and currents at the input (*z*) and output (*z* + d*z*), respectively.

A transmission line is a waveguide system that conveys an electrical signal from one point to another. The transmission line is configured with two or more electrical conductors, as shown in Figure 1. An equivalent circuit model of the transmission line comprises a series resistance (*R*) in Ω/m, series inductance (*I*) in H/m, shunt conductance (*G*) in S/m, and shunt capacitance (*C*) in F/m. The *R*, *L*, *G*, and *C* are affected by the geometry of the transmission line and by electrical properties of dielectric materials and conductors. *R* is an electrical quantity depicting its opposition to the current flow and accounts for the electrical energy loss due to an imperfect conductor. *L* accounts for the magnetic energy of the signal stored in the transmission line that opposes a change in the current. *G* is the inverse quantity of *R* and accounts for the electrical energy loss due to the shunt current leaking into dielectric materials around the transmission line. *C* arises between conductors of the transmission line due to their potential difference and accounts for the capacity to store charges between conductors.

### 2.2. Electromagnetic Wave Propagation on the Transmission Line

Differential equations governing the propagation of electromagnetic waves in the transmission line are referred to as the transmission line or telegrapher’s equations. Applying Kirchoff’s voltage and current laws to the transmission line model shown in Figure 1, and taking the limit as Δ*z* → 0, the transmission line equation becomes [21]:

(1) $$\frac{\partial V(z,\;t)}{\partial z}=-RI(z,\;t)-L\frac{\partial I(z,\;t)}{\partial t}$$

(2) $$\frac{\partial I(z,\;t)}{\partial z}=-GV(z,\;t)-C\frac{\partial V(z,\;t)}{\partial t}$$

where *V* is the voltage in volts, *I* is the current in amperes, and *t* and *z* represent the time and length, respectively. Assuming a harmonic (or sinusoidal) time dependence under a steady-state condition, the cosine-based phasor forms of Equations (1) and (2) are given as follows:

(3) $$\frac{dV_{s}(z)}{dz}=-(R+j\omega L)I_{s}(z)$$

(4) $$\frac{dI_{s}(z)}{dz}=-(G+j\omega C)V_{s}(z)$$

respectively, where *j* is the imaginary number, *ω* is the angular frequency, vs. is the phasor voltage, and *I_s_* the phasor current. Eliminating *I_s_*(*z*) in Equations (3) and (4) yields the wave equation for *V_s_*(*z*):

(5) $$\frac{d^{2}V_{s}(z)}{dz^{2}}-\gamma^{2}V_{s}(z)=0$$

Similarly,

(6) $$\frac{d^{2}I_{s}(z)}{dz^{2}}-\gamma^{2}I_{s}(z)=0$$

where *γ* is the complex propagation constant, which is the function of frequency and is expressed as follows:

(7) $$\gamma =\alpha +j\beta =\sqrt{\left(R+j\omega L)(G+j\omega C\right)}$$

where *α* is the attenuation constant in Nepers per meter, and *β* is the phase constant in radians per meter. The solutions of the linear homogeneous differential equations, Equations (5) and (6), can be found as follows:

(8) $$V_{s}(z)=V_{0}^{+}e^{-\gamma z}+V_{0}^{-}e^{+\gamma z}$$

(9) $$I_{s}(z)=I_{0}^{+}e^{-\gamma z}+I_{0}^{-}e^{+\gamma z}$$

where *V*_0_^+^, *V*_0_^−^, *I*_0_^+^, and *I*_0_^−^ are wave amplitudes, and *e*^−*γx*^ and *e*^+*γz*^ terms represent the forward (same) and backward (opposite) wave propagation directions, respectively, as compared with the generated (incident) wave. It should be noted that the reflected wave at the discontinuity propagates backward. The ratio of amplitudes of the voltage and current of a single wave in the transmission line is defined as the characteristic impedance (*Z*_0_):

(10) $$Z_{0}=\frac{V_{0}{}^{+}}{I_{0}{}^{+}}=-\frac{V_{0}{}^{-}}{I_{0}{}^{-}}=\sqrt{\frac{R+j\omega L}{G+j\omega C}}$$

According to the electromagnetic theory based on Maxwell’s equations, the velocity of electromagnetic waves (*v_p_*) propagating on the transmission line is given as follows [22]:

(11) $$v_{p}=\frac{v_{c}}{\sqrt{\mu \varepsilon /\mu_{0}\varepsilon_{0}}}=\frac{v_{c}}{\sqrt{\mu_{r}\varepsilon_{r}}}$$

where *v_c_* is electromagnetic wave velocity in vacuum (2.998 × 10^8^ m/s), *μ* and *μ*_0_ are the magnetic permeabilities of a specific material and vacuum, respectively, *ε* and *ε*_0_ are the electrical permittivities of a specific material and vacuum, respectively, *μ_r_* is the relative permeability expressed as the ratio of *μ* and *μ*_0_, and *ε_r_* is the relative permittivity or dielectric constant expressed as the ratio of *ε* and *ε*_0_. Most soils are nonmagnetic materials, whose *μ* values are very close to *μ*_0_. Hence, *μ_r_* is very close to unity [23,24]. In dispersive or lossy materials, the measured *ε_r_* in the transmission line is a function of both the energy storage of the dielectric material and losses due to electrical conductivity and/or dielectric relaxation phenomena [25]. The *ε_r_* measured in dispersive materials is expressed as follows [26]:

(12) $$\varepsilon_{r}=\frac{\varepsilon^{\prime }(\omega )}{2}\left[1+\sqrt{1+{\left[\left(\varepsilon^{\prime \prime }(\omega )+\frac{\sigma_{dc}}{\omega \varepsilon_{0}}\right)/(\varepsilon^{\prime }(\omega ))\right]}^{2}}\right]$$

where *ε*′ is the real part of the permittivity associated with energy storage, *ε*″ is the imaginary part of the permittivity associated with relaxation losses, and *σ_dc_* is the electrical conductivity. *σ_dc_* also contributes to the energy loss mechanism (referred to as conduction loss), which is inversely proportional to the frequency [27]. As a function of electrical permittivity, magnetic permeability, and relative permittivity, the characteristic impedance can also be expressed as follows [28,29]:

(13) $$Z_{0}=\sqrt{\frac{\mu_{0}}{\varepsilon_{0}\varepsilon_{r}}}$$

The reflection of electromagnetic waves in the transmission line occurs due to the difference in an electrical impedance. The amplitude ratio of incident and reflected electromagnetic waves is defined as the reflection coefficient (*ρ*):

(14) $$\rho =\frac{V_{r}}{V_{i}}=\frac{I_{r}}{I_{i}}=\frac{-Z_{0}+Z_{1}}{Z_{0}+Z_{1}}$$

where *V_r_* and *V_i_* are amplitudes of reflected and incident electromagnetic waves in volts, respectively, *I_r_* and *I_i_* are amplitudes of reflected and incident electromagnetic waves in amperes, respectively, *Z*_0_ is the characteristic impedance (or instantaneous impedance at medium 0), and *Z*_1_ is the instantaneous impedance at medium 1. If *Z*_1_ > *Z*_0_, electromagnetic waves are reflected with a positive sign. If *Z*_1_ < *Z*_0_, electromagnetic waves are reflected with a negative sign. Impedance inversely depends on the dielectric constant of the medium, as described in Equation (13). If the dielectric constant of medium 0 is greater than that of medium 1, electromagnetic waves will be reflected with a positive sign. However, if the dielectric constant of medium 0 is less than that of medium 1, the reflection will appear negative.

## 3. Experiments

Two model piles were used in the experiments. Transmission lines were installed in model piles to study the propagation of electromagnetic waves. Different types of transmission lines were configured with electrical wires and reinforcement cages to investigate their suitability to detect necking defects filled with different materials. Necking defects were filled with air; dry sand; wet sands with different gravimetric water contents (θ) of 10%, 20%, and 30%; and clay of 30%. The variations in travel times of electromagnetic waves and sensitivity of electromagnetic waves to detect defects with different materials were investigated.

### 3.1. Transmission Line Configuration

**Figure 2:**
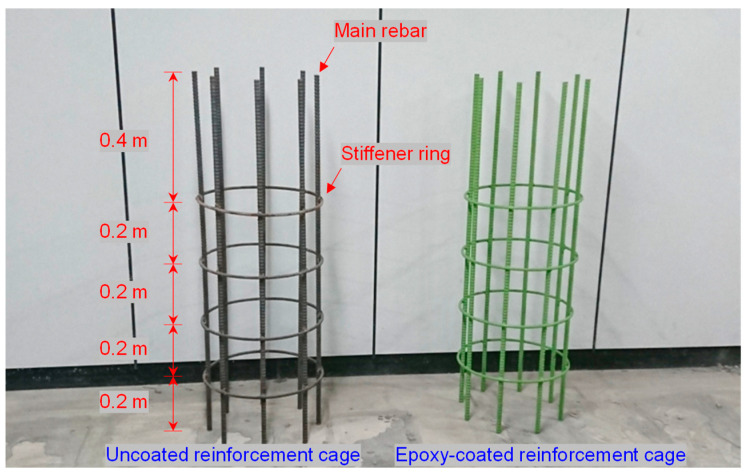
Uncoated reinforcement cage (**left**) and epoxy-coated reinforcement cage (**right**).

Five transmission lines (D1–D5 and T1–T5) were configured with two types of reinforcement cages and four different types of electrical wires. One reinforcement cage was uncoated, whereas the other one was an epoxy-coated reinforcement cage, as shown in Figure 2. The epoxy-coated cage was manufactured at a coating thickness of ~180 μm, in accordance with ASTM A775 [30]. The diameter and height of both reinforcement cages were 0.4 and 1.2 m, respectively, and they comprised eight main rebars and four circular stiffener rings. The diameters of the main rebars and stiffener rings were 16.0 and 10.0 mm, respectively. The main rebars were arranged in a circular array at equal spacing. Stiffener rings were installed at intervals of 0.2 m from the bottom.

**Figure 3:**
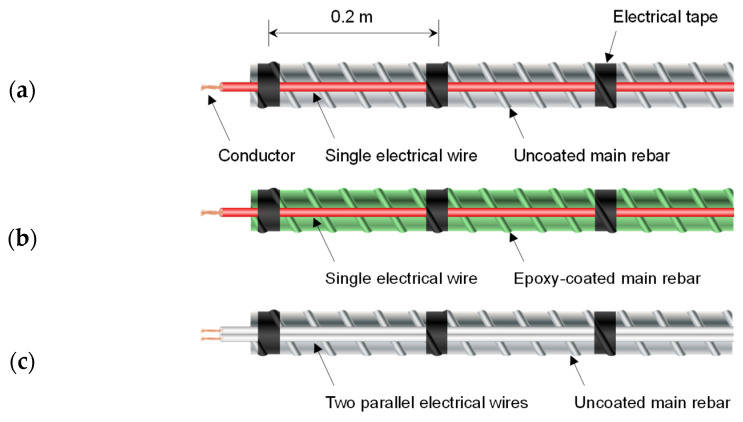

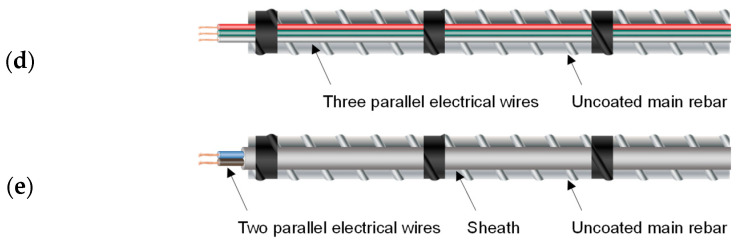
Transmission lines configured with: (**a**) single electrical wire and uncoated main rebar (D1 and T1), (**b**) single electrical wire and epoxy-coated main rebar (D2 and T2), (**c**) two parallel electrical wires (D3 and T3), (**d**) three parallel electrical wires (D4 and T4), and (**e**) two parallel electrical wires wrapped in a sheath (D5 and T5).

The D1 and T1 were two-conductor transmission lines configured with a single electrical wire and main rebar of the uncoated reinforcement cage. The electrical wire was installed alongside the main rebar. The electrical wire comprised a single conductor and insulation, as shown in Figure 3a. The conductor was a flexible stranded annealed copper with a diameter of 2.1 mm. The insulation was polyvinyl chloride (PVC) with a thickness of 0.8 mm. The D2 and T2 were two-conductor transmission lines configured with an electrical wire, which is the same wire used in D1 and T1 and the main rebar of the epoxy-coated reinforcement cage, as shown in Figure 3b. D3 and T3 were two-conductor transmission lines configured with two parallel electrical wires installed alongside the main rebar of the uncoated reinforcement cage, as shown in Figure 3c. The electrical wires comprised a single conductor and insulation. The conductors were flexible stranded annealed coppers with diameters of 1.1 mm. The insulations were PVC with thicknesses of 0.7 mm. D4 and T4 were three-conductor transmission lines configured with three parallel electrical wires installed alongside the main rebar of the uncoated reinforcement cage, as shown in Figure 3d. The conductors were flexible stranded annealed coppers with diameters of diameters of 0.6 mm. The insulation was PVC with thicknesses of 0.4 mm. D5 and T5 were two-conductor transmission lines configured with an electrical cable installed alongside the uncoated reinforcement cage, as shown in Figure 3e. The electrical cable comprised two parallel electrical wires wrapped in one sheath, whereas the electrical wires comprised a single conductor and insulation. The conductors were flexible stranded annealed coppers with diameters of 1.1 mm. The insulations were PVC with thicknesses of 0.5 mm. The sheaths were PVC with thicknesses of 0.6 mm. The electrical wires and cable in D1–D5 and T1–T5 were attached to the main rebars at intervals of 0.2 m using vinyl electrical tape.

### 3.2. Construction of Model Pile

**Figure 4:**
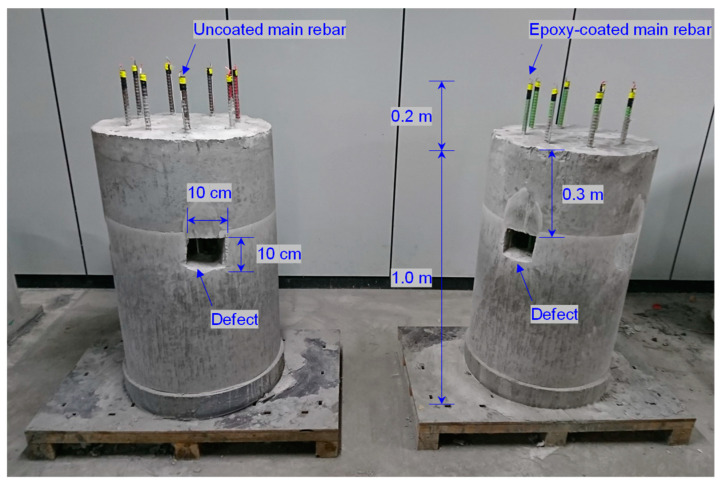
Small-scale model piles constructed with an uncoated reinforcement cage (**left**) and with an epoxy-coated reinforcement cage (**right**).

**Figure 5:**
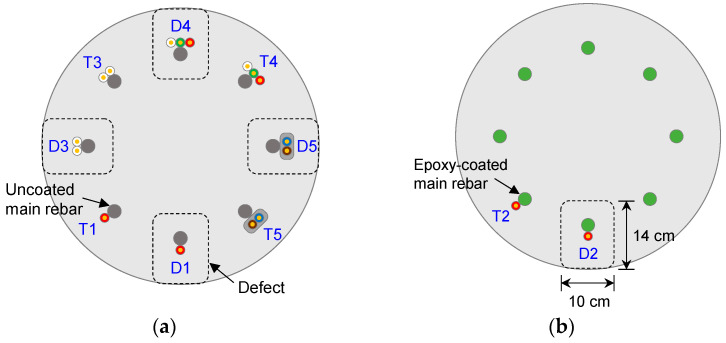
Plan view of model piles constructed with: (**a**) an uncoated reinforcement cage and (**b**) an epoxy-coated reinforcement cage.

Two small-scale model piles with diameters of 0.6 m and lengths of 1.0 m were constructed as shown in Figure 4. One was constructed with the uncoated reinforcement cage, whereas the other was constructed with the epoxy-coated reinforcement cage. The model piles were constructed as follows: the reinforcement cage was inserted into the PVC cylinder with an internal diameter and length of 0.6 and 1.0 m, respectively. The reinforcement cages were positioned at the center of the PVC cylinder. Five necking defects were made of Styrofoam blocks with dimensions of 10 cm × 10 cm × 14 cm; they were located at a depth of 30 cm from the pile head around D1, D3, D4, and D5 in the uncoated reinforcement cage (see Figure 5a) and around D2 in the coated reinforcement cage (see Figure 5b). Cement paste, which is a mixture of cement and water at a weight ratio of 1:0.5 (*w/c* = 50%), was poured into the PVC cylinder, and the PVC cylinder and Styrofoam blocks were removed after curing for 28 days. After curing for 28 days, the dielectric constant of the cement paste was measured using a typical time domain reflectometer (TDR) probe without insulation (CS640-L, Campbell Scientific, Inc., Logan, UT, USA). The dielectric constant of the cement paste was 6.5 under the following conditions: air at a relative humidity of 70–80% and a temperature of ~28 °C. To protect probes from direct contact with materials, two TDR probes (CS640-L) were insulated using a PVC heat-shrink tube. When using TDR probes insulated using PVC with a thickness of either 0.27 or 0.64 mm, the effective dielectric constants of the cement paste were 6.49 and 6.47, respectively. The effective dielectric constants of the cement paste, a result of the combined effects of the cement paste and insulation, were 6.22, 6.15, 4.60, 4.45, and 4.25, as measured using T1–T5 (or D1–D5), respectively, as summarized in Table 1.

To simulate a necking defect caused by soil inclusion, the necking defects in the model piles were filled with soils; these soils included dry and wet silica sands and clay. The dielectric constants of the soils measured using the typical TDR probe without insulation and the effective dielectric constants of the soils (including soils and insulation) measured using transmission lines are summarized in Table 1. The dielectric constants of the dry sand and wet sands with gravimetric water contents of 10%, 20%, and 30%, as measured using the TDR probe, were 1.44, 3.61, 6.87, and 17.30, respectively. The gravimetric water content of the clay was 30%, and the electrical conductivity of clay was 6 dS/m. The dielectric constant of the clay could not be measured, because the signal reflected at the end of the TDR probe was undetectable due to the high electrical conductivity of the clay. It has been reported that the dielectric constant cannot be measured at an electrical conductivity exceeding 3.6 dS/m using a TDR probe without insulation, because the reflected signals are completely attenuated owing to the conduction loss [19,31,32,33,34]. The effective dielectric constants of the dry sand; wet sand with gravimetric water contents of 10%, 20%, and 30%; and clay were 1.54; 3.10, 4.84, and 8.88; and 10.00, respectively, as measured using the TDR probe insulated using PVC with a thickness of 0.27 mm. Using the TDR probe with 0.64-mm-thick PVC insulation, the effective dielectric constants of the dry sand; wet sands with gravimetric water contents of 10%, 20%, and 30%; and clay were 1.69; 2.62, 4.33, and 6.05; and 6.70, respectively.

### 3.3. Generation and Detection of Electromagnetic Waves

**Figure 6:**
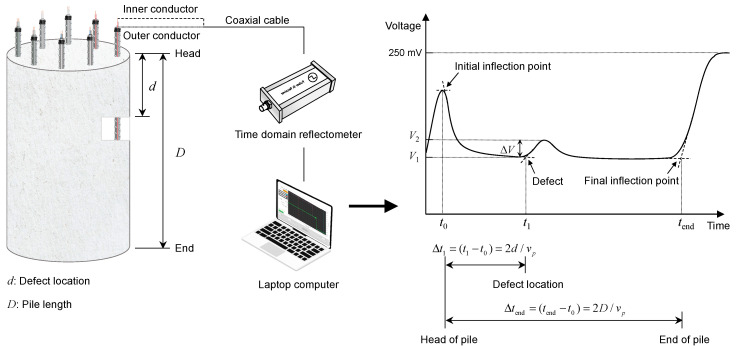
Measurement system.

A time domain reflectometer (TDR) was employed for the generation and detection of electromagnetic waves in model piles. The TDR (HL1101, Hyperlabs, Beaverton, OR, USA) generated a step signal with an amplitude and width of ±250 mV and 3 μs, respectively. The rise time of the step signal was 200 ps between 10% and 90% of its maximum amplitude. The TDR was connected to a transmission line using a coaxial cable, whose characteristic impedance is 50 Ω. For the transmission lines D1 and T1, the inner and outer conductors of the coaxial cable were connected to a single electrical wire and main rebar of the uncoated reinforcement cage, respectively. For D2 and T2, the inner and outer conductors of the coaxial cable were connected to a single wire and main rebar of the epoxy-coated reinforcement cage, respectively. For D3 and T3, the inner and outer conductors of the coaxial cable were connected to one of two parallel electrical wires and the other, respectively. For D4 and T4, the inner and outer conductors of the coaxial cable were connected to the central electrical wire and two side electrical wires, respectively. For D5 and T5, the inner and outer conductors of the coaxial cable were connected to one of two parallel electrical wires wrapped in sheath and the other, respectively. Electrical wires connected to the inner and outer conductors of the coaxial cable were set as a signal path and return path, respectively. The electromagnetic waves reflected from a defect and the end of the model pile were detected by the TDR and, subsequently, recorded by a computer, as shown in Figure 6. In measurements, the noise variance (*σ*^2^) was approximately 1.9, and the signal-to-noise ratio was improved by stacking 200 signals.

### 3.4. Interpretation of the Waveform

The travel time of electromagnetic waves measured using the TDR is typically determined by identifying their inflection points. The dual and single-tangent methods are the most commonly used methods to identify inflection points [35]. In accordance with the dual-tangent method [36], the inflection point is defined as the intersection of two tangent lines to the curve, marked as the point *t*_1_ in Figure 6. The single-tangent method [37], which is also referred to as the flat tangent method, defines the inflection point as the intersection between the horizontally flat and positively sloped tangent lines at the local minimum to the curve, marked as point *t*_end_ in Figure 6. It was reported that the single tangent method is less arbitrary than the dual tangent method [38], and it is, moreover, the most accurate method [39,40]. It was reported, however, that the dual tangent method shows better accuracy for materials with high electrical conductivity. In this study, the single-tangent method was employed to identify inflection points for an accurate analysis. However, the dual-tangent method was adopted when the measured signals were distorted due to conductive materials. The travel time of electromagnetic waves reflected from the defect (Δ*t*_1_) was calculated as the difference between *t*_1_ (time at the first inflection point) and *t*_0_ (time at the initial inflection point). The travel time of electromagnetic waves reflected from the end of the pile (Δ*t*_end_) was calculated as the difference between *t*_end_ (time at the final inflection point) and *t*_0_. The velocity of the electromagnetic wave (*v_p_*) in the model pile was calculated by the ratio of twice the length of the model pile to the travel time (Δ*t*_end_).

In this study, the sensitivity of the transmission lines to a defect detection was evaluated by comparing the amplitude of electromagnetic waves reflected from the defect with that of electromagnetic waves before the reflection. The sensitivity (*S*_T_) was defined as follows:

(15) $$S_{T}=\frac{V_{2}-V_{1}}{V_{\mathrm{input}}}=\frac{\Delta V}{V_{\mathrm{input}}}$$

where *V*_1_ denotes the minimum amplitude of the signal before the reflection, *V*_2_ denotes the peak amplitude of the signal reflected from the defect, *V*_input_ is the input voltage of 250 mV, and Δ*V* is the amplitude difference between the peak (*V*_2_) and minimum (*V*_1_).

### 3.5. Experimental Results

#### 3.5.1. Transmission Lines without Defects

**Figure 7:**
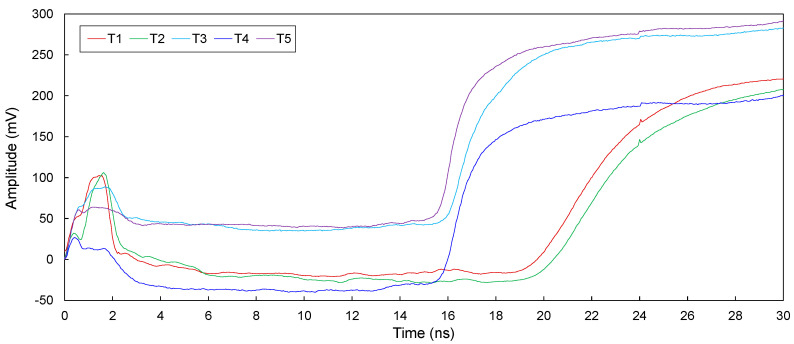
Measured signals for the intact transmission lines (T1–T5).

The electromagnetic waves in transmission lines (T1, T2, T3, T4, and T5) without defects configured in model piles were measured and are plotted in Figure 7. Figure 7 shows that electromagnetic waves reflected from the ends of model piles are clearly detected. For T1, the travel time (Δ*t*_end_) of electromagnetic waves reflected from the end of the model pile is 17.94 ns. For T2–T5, the values of Δ*t*_end_ are 17.91 ns, 14.30 ns, 14.07 ns, and 13.74 ns, respectively, as summarized in Table 2. The Δ*t*_end_ for T1 is slightly greater than that for T2, and both these values are considerably greater than those for T3–T5. Furthermore, the Δ*t*_end_ for T3 is less than that for T4, with the Δ*t*_end_ for T5 being the lowest.

#### 3.5.2. Transmission Lines with Defects

**Figure 8:**
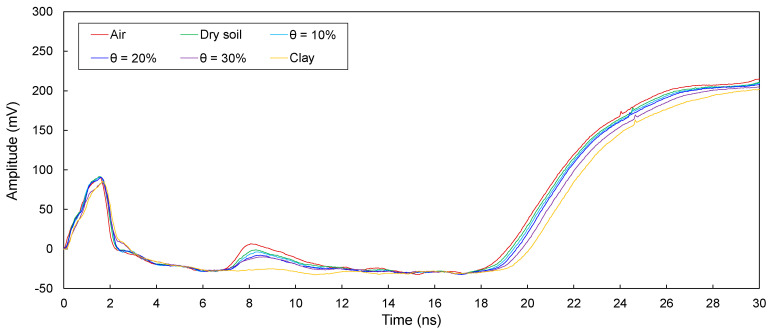
Measured signals for D1 with the defect filled with different materials of air; dry sand; wet sands with θ = 10%, 20%, and 30%; and clay.

**Figure 9:**
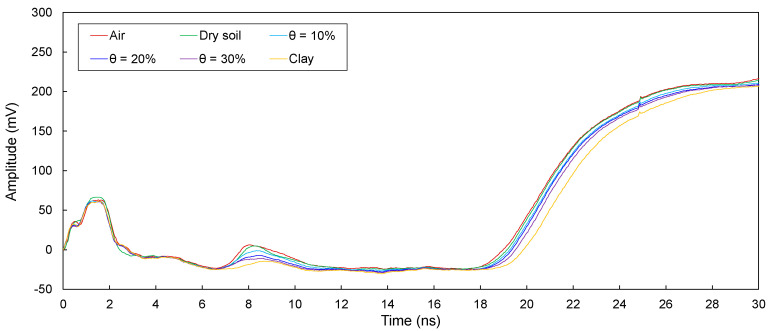
Measured signals for D2 with the defect filled with different materials of air; dry sand; wet sands with θ = 10%, 20%, and 30%; and clay.

**Figure 10:**
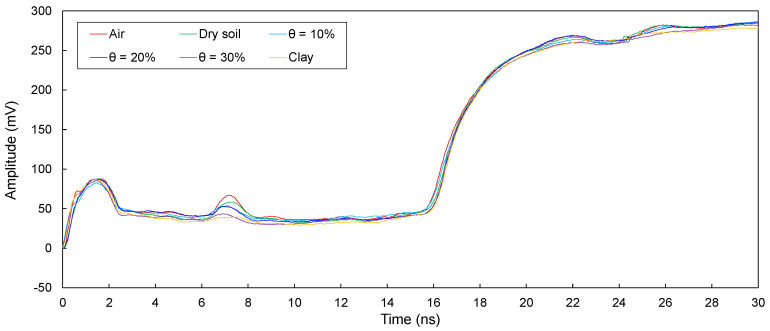
Measured signals for D3 with the defect filled with different materials of air; dry sand; wet sands with θ = 10%, 20%, and 30%; and clay.

**Figure 11:**
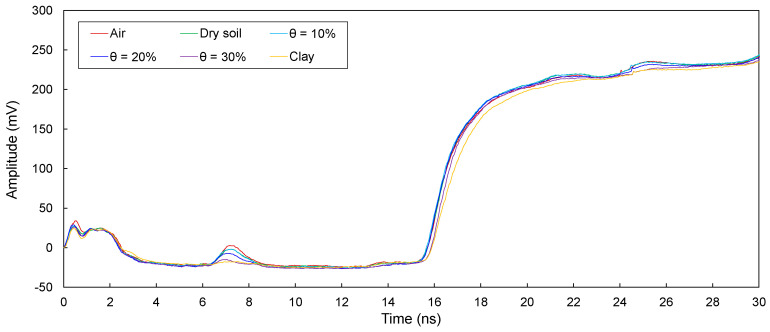
Measured signals for D4 with the defect filled with different materials of air; dry sand; wet sands with θ = 10%, 20%, and 30%; and clay.

**Figure 12:**
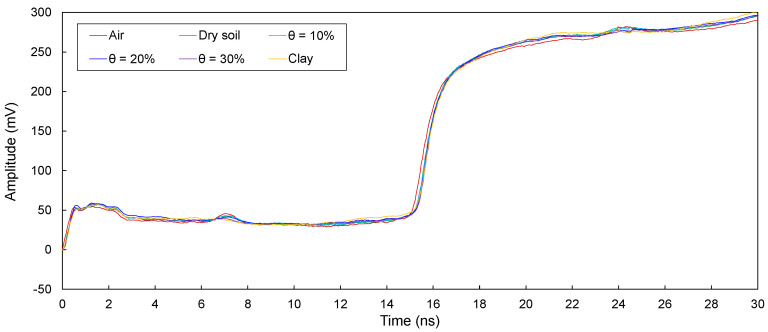
Measured signals for D5 with the defect filled with different materials of air; dry sand; wet sands with θ = 10%, 20%, and 30%; and clay.

The electromagnetic waves in transmission lines (D1, D2, D3, D4, and D5) configured around defective sections in model piles were measured and are plotted in Figure 8, Figure 9, Figure 10, Figure 11 and Figure 12. The travel time of electromagnetic waves reflected from the end of a model pile (Δ*t*_end_) and defect (Δ*t*_1_) were investigated. In addition, for the sensitivity calculation, the amplitude differences (Δ*V*) were determined. The Δ*t*_end_ values for D1–D5 are summarized in Table 2. The values of Δ*t*_1_ and Δ*V* for D1–D5 are summarized in Table 3.

##### Transmission Line D1

Figure 8 shows the measured signals for D1. As shown, the signals reflected from the end of the pile were clearly detected. For all cases, Δ*t*_end_ remains in the range of 16.91–17.67 ns. The signals reflected from the defect of the pile appear under the conditions where defects are filled with air and dry and wet sands. However, a signal reflected from the defect is not detected under the condition where the defect is filled with clay. For conditions where defects are filled with air and dry and wet sands, Δ*t*_1_ is 5.44–5.59 ns, and Δ*V* is in the range of 17.10–34.75 mV.

##### Transmission Line D2

The measured signals for D2 are shown in Figure 9. As shown, the signals reflected from both the end and the defect were clearly detected. The Δ*t*_end_ values range from 16.75 to 17.49 ns. In addition, Δ*t*_1_ is in the range of 5.19–5.42 ns, and Δ*V* ranges from 10.82 to 30.59 mV.

##### Transmission Line D3

The measured signals for D3 are plotted in Figure 10. As shown, the signals reflected from both the end and defect were observed. Δ*t*_end_ ranges from 13.92 to 14.25 ns. In addition, Δ*t*_1_ is 4.45–4.54 ns, and Δ*V* is in the range of 5.12–26.55 mV.

##### Transmission Line D4

Figure 11 shows the measured signals for D4. As shown, the signals reflected from both the end and the defect appeared. Δ*t*_end_ is in the range of 13.53–13.75 ns. In addition, Δ*t*_1_ ranges from 4.38 to 4.50 ns, while Δ*V* is 4.04–23.26 mV.

##### Transmission Line D5

The measured signals for D5 are shown in Figure 12. As shown, the signals reflected from the end were clearly detected. In addition, the signals reflected from the defects filled with air and dry and wet sands were observed. However, a signal reflected from the defect filled with clay was not detected. For all cases, Δ*t*_end_ ranges from 12.91 to 13.04 ns. Under the conditions where the defects are filled with air and dry and wet sands, Δ*t*_1_ is in the range of 4.00–4.05 ns, and Δ*V* ranges from 4.03 to 10.57 mV.

For D1−D5, the electromagnetic waves reflected from the defects and ends of the model piles clearly appeared. Δ*t*_end_ is the lowest for the defects filled with air, followed by those for the defects filled with dry sand and wet sands, respectively. In addition, Δ*t*_end_ increases with an increase in the water content. Furthermore, Δ*t*_end_ values corresponding to the defects filled with clay are the highest. The Δ*t*_end_ values for D1−D5 are less than those for T1–T5. For D1, the Δ*t*_end_ values are slightly greater than those for D2. In addition, Δ*t*_end_ values for D1 and D2 are considerably greater than those for D3−D5. The value of Δ*t*_end_ for D3 is greater than that for D4. Finally, the Δ*t*_end_ for D5 is the lowest. Moreover, for D1−D5, the Δ*t*_1_ values remain similar regardless of the filled material in the defects.

For D1−D5, the Δ*V* values for the defects filled with air are the largest, followed by those for the defects filled with dry sand and wet sands, respectively. In addition, Δ*V* decreases with an increase in the water content. Δ*V* is the lowest when the defects are filled with clay. Furthermore, the value of Δ*V* for D2 is the largest, followed by those for D3 and D4. For D1 and D5, however, Δ*V* values for the defects filled with clay were not estimated, because reflected signals at the defects were not detected.

## 4. Analyses and Discussions

### 4.1. Velocity of Electromagnetic Waves

**Figure 13:**
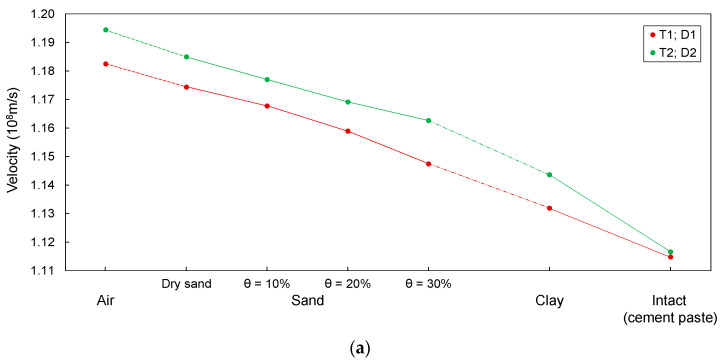

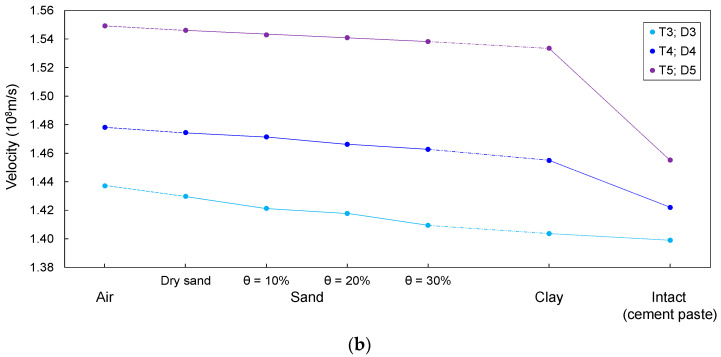
Variation in the velocity of electromagnetic waves according to different materials filling defects: (**a**) T1; D1 and T2; D2 and (**b**) T3; D3, T4; D4, and T5; D5.

The velocity of electromagnetic waves (*v_p_*) in transmission lines configured in model piles were calculated by the ratio of round-trip travel distance (2∙*D*) to travel time (Δ*t*_end_), as described in Figure 6. The calculated values of *v_p_* are summarized in Table 2 and plotted in Figure 13.

#### 4.1.1. Effective Dielectric Constant

The effective dielectric constant depends on the type of transmission lines, as summarized in Table 1, because the sample area is related to the electric potential distribution around conductors. The sample area is defined as the influencing area where materials affect the sensor responses; changes in the material properties outside the sample area do not significantly affect the responses. The sample area of a sensor is dependent on the separation, diameter, number, and insulation of the conductors [20]. For a general, uncoated, two-conductor transmission line, the sample area increases with an increase in separation and slightly increases with respect to the diameter of the conductor. In contrast, the sample area of an uncoated three-conductor transmission line increases with a decrease in the diameter of the conductor. At the same diameter and separation, the two-conductor transmission line has a larger sample area than the three-conductor transmission line. Insulation also affects the sample area of the sensors. For a coated (or insulated) two-conductor transmission line, the sample area increases with a decrease in insulation thickness and an increase in the dielectric constant of the insulation, along with an increase in conductor separation [20,41]. In contrast to the coated two-conductor transmission line, the sample area of a coated three-conductor transmission line increases with a decrease in conductor separation or an increase in the conductor diameter. Nevertheless, the sample area of the coated two-conductor is larger than that of the coated three-conductor transmission line.

In air, the dielectric constant measured using the TDR probe without insulation is less than the effective dielectric constants measured using TDR probes with PVC insulation, because the dielectric constant of the PVC insulation is 2.8−3.8 [42,43]. In addition, the effective dielectric constant increases with an increase in the insulation thickness, because the electrical field affected by the PVC insulation increases. Similarly, the effective dielectric constants measured using transmission lines T1–T5 are greater than the effective dielectric constants measured using the TDR probe with insulation, as shown in Table 1, owing to the conductor separation. It should be noted that the spacing between the rods of the TDR probe is greater than that between the transmission lines. The effective dielectric constant with T1 is slightly smaller than that with T2 owing to the epoxy coating (*ε_r_* ≈ 5) with a thickness of ~180 μm. The effective dielectric constant of T3 is greater than that of T2, because the insulation thickness of T3 is greater than that of T2. The effective dielectric constant of T3 is less than that of T4, because the same area of a three-conductor transmission line is concentrated around the central conductor. The effective dielectric constant of air, as measured using T5, is the greatest, owing to the sheath surrounding the electrical wires.

The effective dielectric constants of the cement paste, as measured using TDR probes with PVC insulation and transmission lines, are less than the dielectric constant of cement paste (*ε_r_* = 6.50) measured using a TDR probe without insulation. The dielectric constant of the PVC insulation is smaller than that of the cement paste. Thus, the insulation decreases the effective dielectric constant of the cement paste.

The dielectric constant of dry sand (*ε_r_* = 1.44) is less than the dielectric constant of the PVC insulation. Thus, the effective dielectric constants measured using the TDR probe with PVC insulation and the transmission lines are greater than the dielectric constant measured using an uninsulated TDR probe. The effective dielectric constants of wet sands and clay were smaller than those of the cement paste, although the actual dielectric constants of wet sands and clay were larger than those of the cement paste, because the insulation (or coating) reduced the sample area. Thus, the effective dielectric constants of wet sands and clay were more affected by the dielectric constant of the insulation than that of the wet sands and clay. Ferré et al. [20] showed that the sample area for a probe with insulation is limited to a very small region adjacent to the probe if the dielectric constant of the soil is greater than 15 or its water content is greater than 28%.

#### 4.1.2. Transmission Lines without Defects

T2 is configured with the single electrical wire and the epoxy-coated rebar in contrast to T1, which is configured with the single electrical wire and uncoated rebar. However, the thickness of the epoxy coating, which is approximately 180 μm, is extremely thin. Thus, the presence of the epoxy coating has a minor effect on the sample area. Therefore, the effective dielectric constants for T1 are slightly larger than those for T2. In contrast, the insulation thicknesses of T3–T5 are significantly larger than those of T1 and T2. This causes reductions in the sample areas of T3–T5. Specifically, the effects of cement paste on the effective dielectric constants for T1 and T2 are greater than that for T3–T5. It should be noted that the dielectric constants of the PVC insulations are in the range of 2.8–3.8 [42,43], which are smaller than those of the cement paste. Thus, the effective dielectric constants for T3–T5 are less than those for T1 and T2. Similarly, the effective dielectric constants for T5 are smaller than those for T3 and T4, owing to the differences in insulation thickness. T4 is the three-conductor transmission line, the sample area of which is smaller than that of T3 (the two-conductor transmission line). Thus, the effective dielectric constants for T4 are less than those for T3, although the insulation thickness of T4 is slightly lower than that of T3.

The velocities of the electromagnetic waves in T1–T5 are 1.117 × 10^8^, 1.115 × 10^8^, 1.399 × 10^8^, 1.422 × 10^8^, and 1.455 × 10^8^ m/s, respectively, as shown in Figure 13. Their velocities are significantly lower than the electromagnetic wave velocity in air (*v_p_* ≈ 3.0 × 10^8^ m/s). This is because the velocity of electromagnetic waves is inversely proportional to the dielectric constant of the materials, as described in Equation (11). T1–T5 are surrounded by the cement paste, which is significantly larger than the dielectric constant of air. Thus, the velocities in T1–T5 are lower than that in air. The velocity of T1 is the lowest, followed by those of T2, T3, T4, and T5, because the effective dielectric constant of T1 is the largest, followed by those of T2, T3, T4, and T5, respectively, as summarized in Table 1. Furthermore, as copper is more conductive than the rebar, the velocities in T1 and T2 are considerably lower than those in T3–T5.

#### 4.1.3. Transmission Lines with Defects

The velocities in transmission lines with a defect filled with air are the greatest, followed by those in transmission lines with defects filled with dry sand and wet sands, respectively, as shown in Figure 13. This variation in velocity is due to the difference in the dielectric constants between materials, and the velocity is inversely proportional to the dielectric constant of a material. Notably, the effective dielectric constants of air were the smallest, followed by those for dry sand and wet sands, respectively, as described in Table 1. In addition, the velocities under conditions where defects are filled with wet sands decrease with an increase in the water content. The dielectric constant of water is generally 78.3 at 25 °C [44], which is significantly larger than that of dry sand. Therefore, the velocity decreases with an increase in the water content. Indeed, the effective dielectric constants of the wet sands increased with an increase in the water content. The velocity for the clay-filled defect was lower than that for the defect filled with wet sand, because the effective dielectric constant of clay is higher than that of wet sand. The velocities are the lowest when the bored piles are intact, because the velocity in a transmission line without defects depends on the effective dielectric constant of the cement paste, whereas that in a transmission line containing a defect depends on the effective dielectric constant of the material filling the defect. Notably, the effective dielectric constant of the cement paste was the largest, as described in Table 1.

The results of this study show that the velocities in T1–T5 and D1–D5 depend on transmission line geometries and the properties of surrounding materials. This phenomenon has been observed in inhomogeneous materials. Moret et al. [45] reported that the dielectric constant differs according to the geometry of a three-rod TDR probe, including the probe spacing and diameter. Hence, further research is required to establish a general equation for describing the relationship between the transmission line geometry and electromagnetic wave velocity in bored piles.

In this study, all necking defects in the model pile had the same length. Thus, the velocities of electromagnetic waves in the defective model piles were only dependent on the geometries of the transmission lines and dielectric constants of the soils. However, the velocity of the electromagnetic waves is affected by the length of the defect. If the defect is filled with air, the velocity will increase as the defect length increases. If the defect is filled with soil with a dielectric constant greater than that of concrete, the velocity decreases as the defect length increases.

### 4.2. Sensitivity

**Figure 14:**
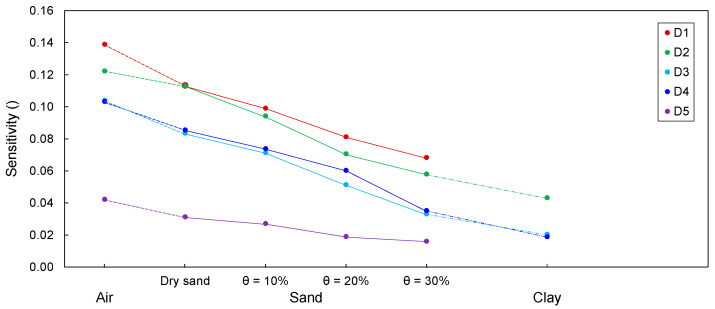
Variation in sensitivity according to different materials filling defects.

The sensitivities were evaluated in accordance with Equation (15) and are plotted and summarized in Figure 14 and Table 3, respectively. Figure 14 shows that the sensitivities of D1–D5 with defects filled with air are larger than those with defects filled with dry sand. The sensitivities were found to decrease with an increase in the water content. Furthermore, the sensitivities for the defects filled with clay were the lowest for all cases. The variation in sensitivity results from the impedance change caused by the difference in the effective dielectric constant. As summarized in Table 1, the effective dielectric constant of air is less than that of dry sand, and the effective dielectric constant of wet sand is greater than that of dry sand. In addition, the effective dielectric constants increased with an increase in the water content. The effective dielectric constant of clay exceeds that of wet sands. Moreover, the effective dielectric constant of cement paste is the highest. As described in Equation (13), impedance is a function of the dielectric constant. Thus, the difference in the impedance between the defect and the cement paste decreases with an increase in the effective dielectric constants of the materials that filled the defects. This causes a reduction in the sensitivity.

For D1, where the defect was filled with clay, the reflected signal at the defect was completely attenuated, and the sensitivity was not evaluated, although the effective dielectric constant of cement paste exceeds that of clay. The rebar of D1 was not insulated, whereas the electrical wire was wrapped with PVC insulation. Thus, this rebar without insulation may have contributed to the conduction loss. The effectiveness of insulation depends on the insulating material and method. Mojid et al. [33] reported that conduction loss may occur depending on the properties of insulating materials. In addition, fully insulated conductors achieve better energy preservation and yield clear signals in highly conductive materials, as compared to partially insulated conductors [17,33]. Thus, further research is necessary to investigate the effects of insulating materials and methods on the electromagnetic waves in bored piles. Recently, Wilczek et al. [46] demonstrated that electrical conductivity contributes to the reduction in amplitude of reflected signals using numerical simulations. It is necessary to perform numerical simulations on transmission lines in soils with various electrical properties to improve sensitivity. For D5, where the defect was filled with clay, the sensitivity was not evaluated because of the minor difference in the impedance between cement paste and clay. It should be noted that the effective dielectric constant for D5 with clay is similar to that of cement paste, as summarized in Table 1.

As shown in Figure 14, the sensitivity of D1 was the highest, followed by those of D2, D3, D4, and D5, respectively, because of the impedance change caused by the differences in the effective dielectric constants of the cement paste and materials that filled the defects. Furthermore, the difference in the effective dielectric constants of the cement paste and defect-filling material for D1 is the largest, followed by those for D2, D3, D4, and D5, respectively, as shown in Table 1.

In this study, the variations in sensitivity due to defect size were not considered. However, Chajes et al. [47] reported that sensitivity depends on the defect size. Thus, further research will be required to investigate the influence of the size of defects in bored piles on sensitivity. The skin effect, which is the degree of electromagnetic energy concentrated close to the surface of conductors, reduces the effective cross-section of conductors [48]. Thus, the effective resistance of conductors may increase due to the skin effect, which may deteriorate the sensitivity. The arrangement and geometry of conductors, including their spacing, are the most important factors affecting the skin effect [49]. Therefore, further research on the influence of the skin effect on the resistance of transmission lines in piles is required to improve sensitivity.

### 4.3. Estimated Location of Necking Defect

**Figure 15:**
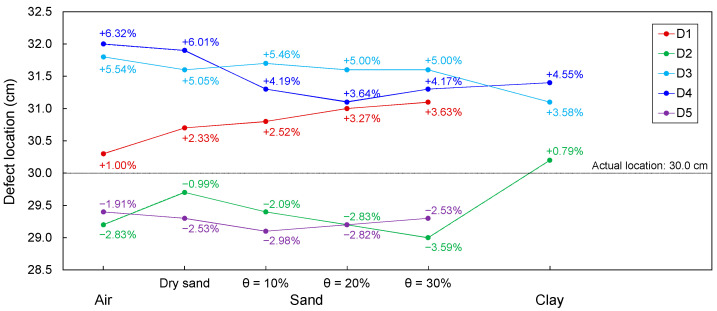
Estimated defect location and error.

A defect location (*d*) was estimated as half the product of the electromagnetic wave velocity (*v_p_*) in the transmission line configured around an intact section in a model pile and the difference in the travel time (Δ*t*_1_) between electromagnetic waves reflected from the head and from the defect, as described in Figure 6. The estimated defect locations are plotted in Figure 15.

The presence of a defect and materials surrounding transmission lines manifest a signal dispersion and attenuation, and signals reflected at defects are therefore distorted, resulting in errors in the determination of the Δ*t*_1_. Indeed, signal distortions are observed in signals reflected from defects, as shown in Figure 8, Figure 9, Figure 10, Figure 11 and Figure 12. However, the effect of signal distortions on the estimation of defect locations was not significant. Although signal distortions caused errors, estimated defect locations for D1–D5 are similar to the actual defect location of 30.0 cm, even though the defects were filled with dry and wet sands and clay. Errors in the estimated defect locations are estimated to range from −3.59% to +6.32%, as shown in Figure 15. This demonstrates that electromagnetic waves can be utilized to estimate a defect location, even if the defect is filled with soils.

## 5. Summary and Conclusions

The objective of this study was to investigate variations in the velocity and sensitivity of electromagnetic waves in different types of transmission lines configured in model piles to detect necking defects filled with soil. The experiments were conducted with defective model piles, wherein five different types of transmission lines were configured, i.e., a two-conductor transmission line with a single electrical wire and uncoated rebar (T1 and D1), two-conductor transmission line with a single electrical wire and epoxy (T2 and D2), two-conductor transmission line with two parallel electrical wires (T3 and D3), three-conductor transmission line with three parallel electrical wires (T4 and D4), and two-conductor transmission line with two parallel electrical wires wrapped with a sheath (T5 and D5). The defects were introduced around D1–D5, whereas areas around T1–T5 were intact. Necking defects were filled with different materials of dry sand; wet sands with water contents of 10%, 20%, and 30%; clay; and air to simulate defects due to soil inclusion. The electromagnetic waves were generated and detected using a TDR. The electrical wires were protected from direct contact with air, water, and soils via insulation. This configuration enabled the permanent monitoring of bored piles, provided the electrical wires remained intact.

The experimental results showed that the velocity in T1 was the lowest, followed by those in T2, T3, T4, and T5, respectively. This was due to the difference in the effective dielectric constant between the transmission lines. It should be noted that T1–T5 were configured with different conductor geometries and insulating properties. Thus, the transmission lines had different sample areas; those of T1 and T2 were larger than those of T3–T5, because the thicknesses of the insulating materials of T3–T5 were greater than those of T1 and T2. The sample area of T1 was slightly larger than that of T2, because the thickness of the epoxy coating was extremely low. Meanwhile, the sample area of T5 was smaller than those of T3 and T4, because T5 was wrapped with thicker insulation. The sample area of T3 was larger than that of T4; however, the insulation thickness of T4 was slightly smaller than that of T3. This is because a two-conductor transmission line has a larger sample area than a three-conductor transmission line. Consequently, the effective dielectric constant for T1 was the largest, followed by those for T2, T3, T4, and T5, respectively. For D1–D5, the velocities of the electromagnetic waves, under the condition of the defect filled with dry sand, were lower than those under the condition where the defect was filled with air. In addition, the velocities in D1–D5 decreased with an increase in the water content. The velocities of electromagnetic waves for the defect filled with clay were the lowest. The variation in the velocity was due to the differences in the effective dielectric constants of the materials that filled the defects. The effective dielectric constants of air were the smallest, followed by those for dry sand, wet sand, and clay, respectively.

For D1–D5, when the defects were filled with dry sand, the sensitivities were lower than those when the defects were filled with air and higher than those when the defects were filled with wet sand. Moreover, the sensitivities were the lowest when the defects were filled with clay. This is due to the impedance change caused by the differences between the effective dielectric constant of the cement paste and those of the materials that filled the defects. However, when the defect was filled with clay, the sensitivity of D1 was not evaluated, because the reflected signal at the defect was completely attenuated, although there was a difference in the effective dielectric constant between the cement paste and clay. This may be because the effectiveness of insulation in reducing conduction loss is dependent on the conductor geometries and properties of the insulating materials.

This study demonstrated that electromagnetic waves can be effectively used to detect necking defects in bored piles with wet and conductive soils. However, the separation and insulation of transmission lines significantly affected the velocity and sensitivity of electromagnetic waves in the model piles. Therefore, an appropriate geometry for a transmission line should be designed to clearly capture the signal reflection and accurately estimate the location of the defect. Moreover, several questions regarding the sensitivity improvement remain unanswered. Further studies should focus on the arrangement, geometry, and insulation of the transmission line; the skin effect on the effective cross-section of conductors should be investigated to minimize an increase in conductor resistance. Additionally, studies are required to investigate the effects of the location, shape, and size of defects on the reflection of electromagnetic wave propagation and to evaluate the long-range propagation of electromagnetic waves in piles.

## Figures and Tables

**Table 1:** Dielectric constants measured using a time domain reflectometer (TDR) probe without insulation and effective dielectric constants measured using TDR probes with polyvinyl chloride (PVC) insulation and transmission lines (T1 and D1–T5 and D5).

TDR Probe and Transmission Line		Air	Cement Paste	Dry Sand	Wet Sand			Clay θ = 30%
No.	Signal (SP) and Return Paths (RP)				θ = 10%	θ = 20%	θ = 30%	
TDR probe ^(1)^	SP: central rod RP: side rods	1	6.50	1.44	3.61	6.87	17.30	N/A ^(4)^
TDR probe ^(2)^	SP: central rod with insulation RP: side rods with insulation	1.10	6.49	1.54	3.10	4.84	8.88	10.00
TDR probe ^(3)^	SP: central rod with insulation RP: side rods with insulation	1.16	6.47	1.69	2.62	4.33	6.05	6.70
T1and D1	SP: electrical wire RP: uncoated rebar	1.50	6.22	1.42	1.99	2.67	3.36	5.32
T2 and D2	SP: electrical wire RP: coated rebar	1.52	6.15	1.74	2.01	2.82	3.51	5.05
T3 and D3	SP: electrical wire RP: electrical wire	2.05	4.60	2.73	2.96	3.17	3.31	3.66
T4 and D4	SP: central electrical wire RP: side electrical wires	2.23	4.45	2.85	3.26	3.46	3.64	3.94
T5 and D5	SP: electrical wire with sheath RP: electrical wire with sheath	2.75	4.25	3.52	3.59	3.84	3.90	4.02

^(1)^ TDR probe without PVC insulation. ^(2)^ TDR probe with PVC insulation of thickness 0.27 mm. ^(3)^ TDR probe with PVC insulation of thickness 0.64 mm. ^(4)^ Dielectric constant was unmeasurable due to conduction loss.

**Table 2:** Travel times of electromagnetic waves reflected from the ends (Δ*t*_end_) and velocities (*v_p_*) in the transmission lines.

Transmission Line		No Defect	Air	Dry Sand	Wet Sand			Clay θ = 30%
					θ = 10%	θ = 20%	θ = 30%	
T1; D1	Δ*t*_end_ (ns)	17.94	16.91	17.03	17.13	17.26	17.43	17.67
	*v_p_* (10^8^ m/s)	1.115	1.182	1.174	1.168	1.159	1.147	1.132
T2; D2	Δ*t*_end_ (ns)	17.91	16.75	16.88	16.99	17.11	17.20	17.49
	*v_p_* (10^8^ m/s)	1.117	1.194	1.185	1.177	1.169	1.163	1.144
T3; D3	Δ*t*_end_ (ns)	14.30	13.92	13.99	14.07	14.11	14.19	14.25
	*v_p_* (10^8^ m/s)	1.399	1.437	1.430	1.421	1.418	1.409	1.404
T4; D4	Δ*t*_end_ (ns)	14.07	13.53	13.56	13.59	13.64	13.67	13.75
	*v_p_* (10^8^ m/s)	1.422	1.478	1.474	1.471	1.466	1.463	1.455
T5; D5	Δ*t*_end_ (ns)	13.74	12.91	12.94	12.96	12.98	13.00	13.04
	*v_p_* (10^8^ m/s)	1.455	1.549	1.546	1.543	1.541	1.538	1.534

**Table 3:** Travel time of electromagnetic waves reflected from defects (Δ*t*_1_), amplitude difference between the peak and minimum (Δ*V*), and sensitivity of signals reflected at the defects.

Transmission Line		Air	Dry Sand	Wet Sand			Clay θ = 30%
				θ = 10%	θ = 20%	θ = 30%	
D1	Δ*t*_1_ (ns)	5.44	5.51	5.52	5.56	5.59	-
	Δ*V* (mV)	34.75	28.33	24.79	20.34	17.10	-
	Sensitivity	0.139	0.113	0.099	0.081	0.068	-
D2	Δ*t*_1_ (ns)	5.23	5.32	5.26	5.23	5.19	5.42
	Δ*V* (mV)	30.59	28.26	23.57	17.68	14.51	10.82
	Sensitivity	0.122	0.113	0.094	0.071	0.058	0.043
D3	Δ*t*_1_ (ns)	4.54	4.52	4.54	4.51	4.51	4.45
	Δ*V* (mV)	26.02	20.89	17.85	12.86	8.30	5.12
	Sensitivity	0.104	0.084	0.071	0.051	0.033	0.020
D4	Δ*t*_1_ (ns)	4.50	4.49	4.40	4.38	4.40	4.42
	Δ*V* (mV)	23.83	21.38	18.47	15.08	8.83	4.04
	Sensitivity	0.103	0.086	0.074	0.060	0.035	0.019
D5	Δ*t*_1_ (ns)	4.05	4.02	4.00	4.01	4.02	-
	Δ*V* (mV)	10.57	7.84	6.79	4.77	4.03	-
	Sensitivity	0.042	0.031	0.027	0.019	0.016	-
